# Folic acid delays development of atherosclerosis in low‐density lipoprotein receptor‐deficient mice

**DOI:** 10.1111/jcmm.13599

**Published:** 2018-03-23

**Authors:** Sunlei Pan, Huahua Liu, Feidan Gao, Hangqi Luo, Hui Lin, Liping Meng, Chengjian Jiang, Yan Guo, Jufang Chi, Hangyuan Guo

**Affiliations:** ^1^ The First Clinical Medical College Wenzhou Medical University Wenzhou China; ^2^ Department of Cardiology Shaoxing People's Hospital Shaoxing Hospital of Zhejiang University Shaoxing China

**Keywords:** atherosclerosis, dedifferentiation, folic acid, LDLR−/− mice, vascular smooth muscle cells

## Abstract

Many studies support the cardioprotective effects of folic acid (FA). We aimed to evaluate the utility of FA supplementation in preventing the development of atherosclerotic in low‐density lipoprotein receptor‐deficient (LDLR−/−) mice and to elucidate the molecular processes underlying this effect. LDLR−/− mice were randomly distributed into four groups: control group, HF group, HF + FA group and the HF + RAPA group. vascular smooth muscle cells (VSMCs) were divided into the following four groups: control group, PDGF group, PDGF + FA group and PDGF + FA + RAPA group. Blood lipid levels, oxidative stress and inflammatory cytokines were measured. Atherosclerosis severity was evaluated with oil red O staining. Haematoxylin and eosin (H&E) staining was used to assess atherosclerosis progression. Immunohistochemical staining was performed with antismooth muscle α‐actin (α‐SMA) antibodies and anti‐osteopontin (OPN) antibodies that demonstrate VSMC dedifferentiation. The protein expression of α‐SMA, OPN and mechanistic target of rapamycin (mTOR)/p70S6K signalling was detected by Western blot analysis. FA and rapamycin reduced serum levels of total cholesterol, triacylglycerol, LDL, inhibiting oxidative stress and the inflammatory response. Oil red O and H&E staining demonstrated that FA and rapamycin inhibited atherosclerosis. FA and rapamycin treatment inhibited VSMC dedifferentiation in vitro and in vivo, and FA and rapamycin attenuated the mTOR/p70S6K signalling pathway. Our findings suggest that FA attenuates atherosclerosis development and inhibits VSMC dedifferentiation in high‐fat‐fed LDLR−/− mice by reduced lipid levels and inhibiting oxidative stress and the inflammatory response through mTOR/p70S6K signalling pathway.

## 1 INTRODUCTION

Atherosclerosis is the leading cause of morbidity and mortality worldwide and is characterized by a chronic inflammatory response, endothelial dysfunction, a build‐up of lipids and a vascular smooth muscle cell (VSMC) phenotypic switch.^1^, ^2^ Recent studies suggest that VSMCs play a central role in the development and progression of atherosclerosis.^3^ During the development of atherosclerosis, VSMCs change from a contractile phenotype to a synthetic phenotype, migrate to the intima, increase their proliferative ability and promote the synthesis of extracellular matrix proteins.^4^, ^5^ VSMCs exhibit a contractile phenotype characterized by the expression of contractile marker such as α‐SMA and synthetic phenotype characterized by the expression of synthetic marker osteopontin (OPN).^3^, ^6^ Therefore, the regulation of the VSMC phenotype may be an alternative strategy for effective atherosclerosis prevention and treatment.

Folic acid (FA) is a water‐soluble vitamin B that is essential for amino acid metabolism, also naming it vitamin B9.^7^ In the past decade, epidemiological studies have shown that FA supplements can prevent neural tube defects,^8^ reduce the risk of megaloblastic anaemia 9 and prevent some malignancies.^10^ It has also been demonstrated that FA has anti‐inflammatory,^11^ anti‐oxidative^12^ and anti‐apoptotic effects.^13^ FA also exhibits a cardioprotective effect,^14^ and it has been reported that dietary supplementation with FA can improve endothelial function.^15^ Additionally, Huo et al^16^ reported that FA supplementation significantly reduced the risk of stroke among hypertensive adults in China without a prior history of stroke or myocardial infarction.

The mammalian target of rapamycin (mTOR) is a serine/threonine kinase that regulates various cellular processes including proliferation, growth, migration and differentiation.^17^ It has been reported that the VSMC switch from a contractile phenotype to synthetic phenotype is associated with mTOR/p70S6K activation in atherosclerotic lesions.^18^, ^19^ However, the regulation of the mTOR/p70S6K signalling pathway is still not fully understood. Therefore, in this study we aimed to determine the ability of FA supplementation to delay the development of atherosclerosis lesions and to analyse the effects of FA on VSMC dedifferentiation through the mTOR/p70S6K signalling pathway in low‐density lipoprotein receptor‐deficient (LDLR−/−) mice.

## 2 MATERIALS AND METHODS

### 2.1 Materials

FA was supplied by Sigma‐Aldrich (St Louis, MO., USA); rapamycin was supplied by AG Scientific, Inc. (San Diego, CA., USA); ELISA kits were purchased from R&D systems (Minneapolis, MN., USA); oxidative stress detection kits were purchased from Jiancheng Bioengineering Institute (NanJing, China); oil red O stain was obtained from Sigma‐Aldrich; Lillie‐Mayer's haematoxylin and eosin (H&E) stain was obtained from Cosmo Bio Company (Tokyo, Japan); Dulbecco's modified Eagle's medium (DMEM, Gibco, USA), cell lysis buffer (Cell signalling technology, USA) and antibodies against α‐smooth muscle actin (SMA), OPN, mTOR, phosphorylated (p)‐mTOR, p70S6K and phosphorylated (p)‐p70S6K were obtained from Cell Signaling (Danvers, MA., USA).

### 2.2 Animal models

Twenty 6‐week‐old male homozygous LDLR−/− mice on C57BL6/J background were purchased from the model animal research centre of Nanjing University (Nanjing, China). Mice were feeding in Shaoxing City People's Hospital experimental animal centre. Following adaptation to their environment for 1 week, the LDLR−/− mice were randomized into four dietary groups as follows: mice fed with a standard diet (NC group), mice fed a high‐fat diet (20% fat, 20% sugar and 1.25% cholesterol) (HF group), mice fed a high‐fat diet with FA supplementation (75 ug/kg/d^20^) (HF + FA group) and mice fed a high‐fat diet with rapamycin (10 mg/kg^21^) (HF + RAPA group). Folic acid was once‐daily oral gavage for 16 weeks; the control and high‐fat groups received saline by oral gavage. The rapamycin group received intraperitoneal injections. The protocol was approved by the ethical committee for animal research of the Shaoxing City People's Hospital. After 16 weeks of treatment, the mice were fasted overnight and killed. Serum was collected, centrifuged at 1200 g for 5 minutes and harvested for determination of serum lipid levels. The heart and aorta were removed and perfused with phosphate‐buffered saline and then for atherosclerotic lesion evaluation, haematoxylin and eosin staining was performed. The mouse aortic root which connects with the heart was isolated and fixed with 4% paraformaldehyde for 12 hour, embedded in paraffin and cut into 5‐μm serial sections. Two aortas were subjected to Western blotting, and the other three were treated with oil red O staining to detect atherosclerosis lesions.

### 2.3 Cell culture

The mice aortic smooth muscle cell line, MOVAS, was obtained from the American Type Culture Collection. The VSMCs were randomly divided into the following groups: control group, PDGF group, PDGF + FA group and PDGF + FA + RAPA group. Before the experiment, VSMCs were serum‐starved for 24 hour in DMEM. VSMCs in the control group were cultured without any treatment. VSMCs in the PDGF‐BB group were treated with PDGF‐BB (20 ng/mL) for 24 hour. VSMCs in PDGF−BB + FA group were treated with PDGF − BB (20 ng/mL) and FA (20 μmol/L) for 24 hour. VSMCs in PDGF−BB + FA + RAPA group were treated with PDGF − BB (20 ng/mL), FA (20 μmol/L) and rapamycin (20 nmol/L) for 24 hour.

### 2.4 Serum biochemical determinations

An automatic biochemistry analyser (Olympus AU2700, Japan) was used to measure serum concentrations of total cholesterol (TC), triglyceride (TG) and high‐density lipoprotein cholesterol (HDL‐C). The level of low‐density lipoprotein cholesterol (LDL‐C) was calculated using the Friedewald formula.^22^

### 2.5 Oxidative stress measurement and inflammatory cytokine detection

Oxidative stress was assessed by detecting malondialdehyde (MDA), superoxide dismutase (SOD) and glutathione peroxidase(GSH‐Px)in the LDLR−/− mouse serum according to the oxidative stress detection kit instructions. The concentrations of inflammatory factors in LDLR−/− mouse serum, including tumour necrosis factor‐α, interleukin‐1β and interleukin‐6 were determined using commercial enzyme‐linked immunosorbent assay (ELISA) kits according to the manufacturer's protocol.

### 2.6 Determination of atherosclerotic plaques in the isolated aorta

After the mice were killed by euthanasia with an intraperitoneal injection of pentobarbital sodium (45 mg/kg), the aorta was harvested and stained with oil red O to assess for the presence atherosclerotic plaques. In brief, stained aortas were photographed using a digital camera (Olympus BX53, Tokyo, Japan). The area occupied by plaques was measured with DP Manager/Controller software (Mitani Co., Tokyo, Japan), and data were analysed with WIN Roof software (Ver. 5.8.1, Mitani Co.). The severity of atherosclerosis was expressed as a percentage of the atherosclerotic plaque area to the total aortic surface area.

### 2.7 Haematoxylin and eosin staining of mouse aortas

The mouse aortic roots were isolated and fixed with 4% paraformaldehyde for 12 hour, embedded in paraffin and cut into 5‐μm serial sections. Aortic sections were stained with Lillie‐Mayer's H&E to evaluate atherosclerotic lesions in the aortic root. The lesion areas were quantified using Image‐Pro Plus 6.0 software (Media Cybernetics).

### 2.8 Immunohistochemistry analysis

The aortic roots were harvested and fixed in 4% paraformaldehyde for 12 hour. The aortic roots were sliced into 5 μm thick for morphometric analyses. Histological sections from the aorta were treated with 3% hydrogen peroxide to block endogenous peroxidase activity, and immunohistochemical staining was performed with anti‐α‐SMA and anti‐OPN antibodies. The positive areas were measured in five non‐overlapping fields with a DP Manager/Controller and Image‐Pro Plus software.

### 2.9 Western blot for α‐SMA, OPN, p‐mTOR, mTOR, p‐p70S6K and p70S6K

Aortas and VSMCs were homogenized in ice‐cold cell lysis buffer plus protease inhibitor cocktail. Protein expression in the aortas and in VSMCs was detected by Western blot analysis using the primary antibodies for α‐SMA, OPN, mTOR, p‐mTOR, p70S6K and p‐p70S6K. Each sample was harvested and separated on 10% sodium dodecyl sulphate‐polyacrylamide gel electrophoresis and electrotransferred onto polyvinylidene fluoride membranes. The membrane was blocked in 5% skim milk for 30 minutes, followed by incubation with the primary antibody overnight at 4°C. The samples were then further incubated with a secondary antibody for 2 hour at room temperature. The bands were detected using an enhanced chemiluminescence reagent, and protein levels were determined by normalization to GAPDH.

### 2.10 Statistical analysis

At least three independent measurements were performed for all assays. All data were reported as the mean ± standard error (SE). Parameters were evaluated by one‐way ANOVA with least significant difference (LSD) post hoc multiple comparison tests. Differences were considered to be significant at *P *< .05.

## 3 RESULTS

### 3.1 Folic acid supplementation ameliorated dysregulated blood lipid metabolism

LDLR−/− mouse serum TC, TG, LDL‐C and very low‐density lipoprotein cholesterol (VLDL‐C) levels in the HF + FA group were significantly lower than those of the HF group after treatment for 16 weeks. HDL‐C levels were significantly increased in the HF + FA group compared to those seen in the HF group (*P *< .05). The HF + RAPA group showed results similar to those of the HF + FA group as shown in Table 1. These findings suggest that FA provided important beneficial cardiovascular protective effects in LDLR−/− mice fed a high‐fat diet. In addition, compared with the HF group,the weight of the HF + FA group and HF + RAPA group did not decrease, suggesting that the decrease in blood lipid level in FA and RAPA was independent of the weight.

**Table 1:** Bodyweight and biochemical parameters in each groups of mice

Parameters	NC	HF	HF + FA	HF + RAPA
Bodyweight(g)	23.37 ± 1.35	27.94 ± 1.07^a^	27.52 ± 0.84	27.67 ± 0.96
TC (mg/dL)	216 ± 15	675 ± 42^a^	481 ± 30^b^	321 ± 29^b^
TG (mg/dL)	120 ± 6	235 ± 7^a^	186 ± 6^b^	143 ± 8^b^
LDL‐C (mg/dL)	139 ± 12	600 ± 35^a^	420 ± 17^b^	273 ± 25^b^
VLDL‐C (mg/dL)	26 ± 3	45 ± 2^a^	39 ± 3^b^	32 ± 2^b^
HDL‐C (mg/dL)	60 ± 4	30 ± 3^a^	37 ± 5^b^	45 ± 4^b^

TC, total cholesterol; TG, triacylglycerol; LDL‐C, low‐density lipoprotein; VLDL‐C, very low‐density lipoprotein cholesterol; HDL‐C, high‐density lipoprotein; FA, folic acid.

The values are expressed as the mean ± standard error of the mean (n = 5).

a *P *<* *.05 vs the NC group.

b *P *<* *.05 vs the HF group.

### 3.2 Folic acid supplementation decreased oxidative stress and inflammation

**Figure 1:**
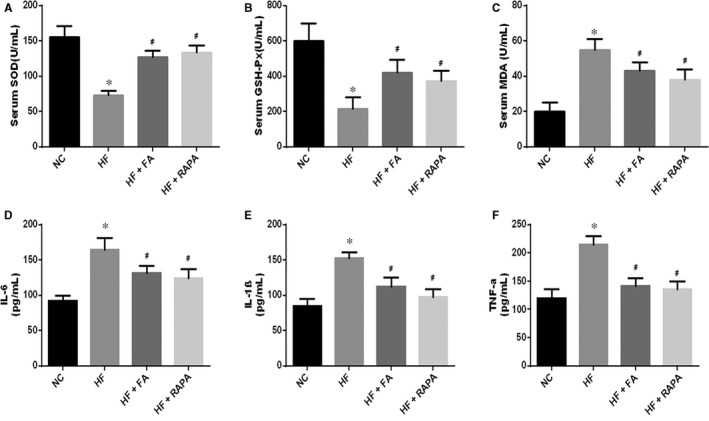
Effect of folic acid on the levels of oxidative stress and inflammation. A, Serum superoxide dismutase (SOD) level; B, serum glutathione peroxidase(GSH‐Px) level; C, malondialdehyde (MDA) level; D, serum interleukin (IL)‐6 level; E, serum IL‐1β level; F, serum tumour necrosis factor‐α (TNF‐α) level. The values are expressed as the mean ± standard error of the mean (n = 5). *P < .05 vs the NC group; ^#^ *P* < .05 vs the HF group

The effects of folic acid supplementation on markers of oxidative stress and inflammation were analysed. In the HF + FA and HF + RAPA groups, the levels of SOD and GSH‐Px were significantly higher than those seen in the HF group (Figure 1A, B and C). However, MDA levels, which are a marker of oxidative damage, were decreased in the HF + FA and HF + RAPA groups. Moreover, in the HF + FA and HF + RAPA groups, IL‐6, IL‐1β and TNF‐α levels were reduced compared with the levels seen in the HF group (Figure 1D, E and F). These results demonstrate that FA supplementation suppressed oxidative stress and inflammation in the high‐fat‐fed LDLR−/− mice.

### 3.3 Folic acid supplementation suppressed atherosclerosis plaque progression

**Figure 2:**
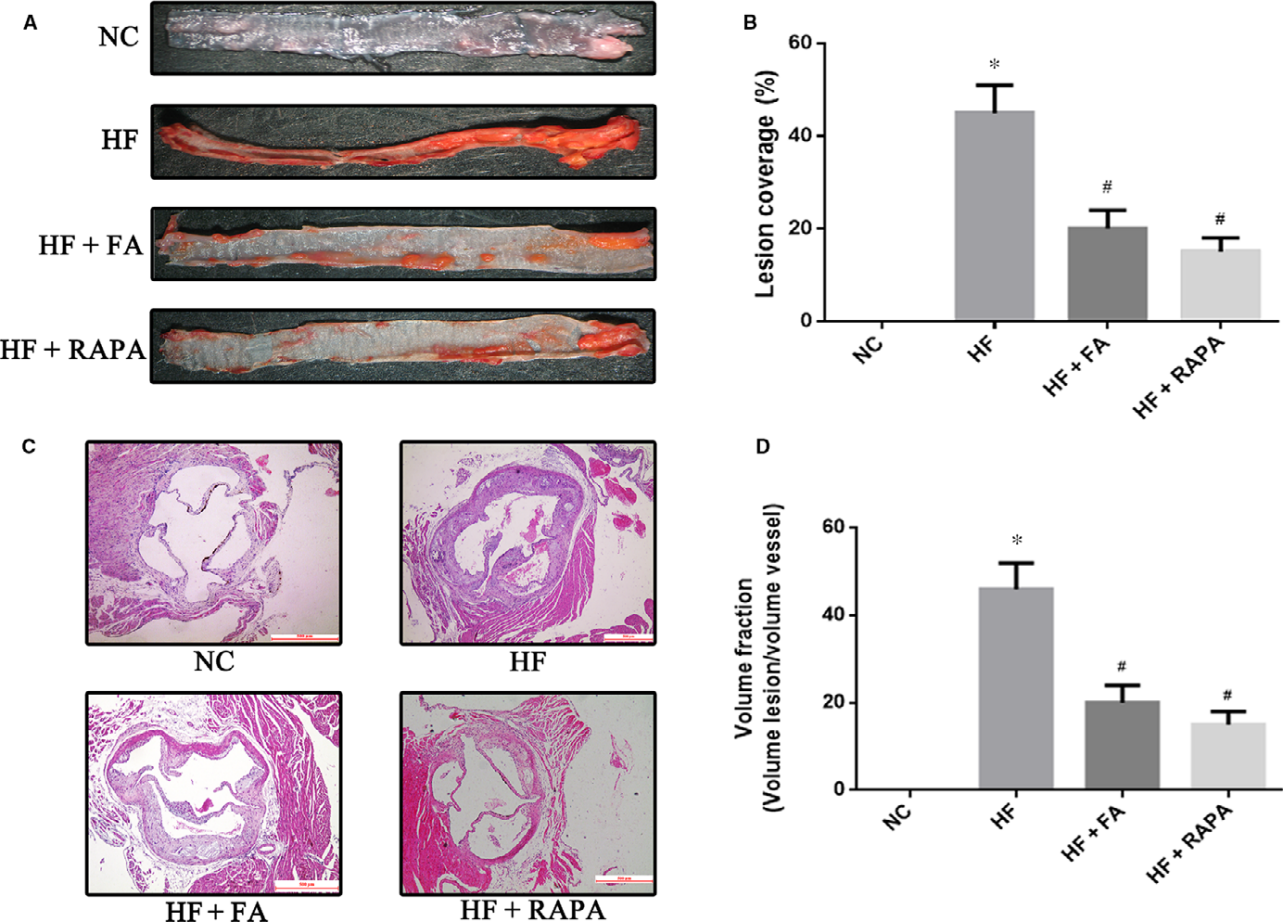
Effect of folic acid on atherosclerotic lesion area. (A) (B) Atherosclerotic lesions shown with oil red O staining and quantitative analysis of atherosclerotic lesion area (n = 3 for each group). *P < .05 vs the NC group; ^#^ *P* < .05 vs the HF group. (C) (D) representative images and quantification of haematoxylin and eosin staining of the aortic sinus. Scale bar = 500 μm. The values are expressed as the mean ±standard error of the mean (n = 5 for each group). *P < .05 vs the NC group; ^#^ *P* < .05 vs the HF group

Atherosclerotic lesions were stained using oil red O stain to determine the area of atherosclerosis in each study group. As shown in Figure 2A and B, oil red O staining revealed that LDLR−/− mice fed a high‐fat diet had a significantly increased area of atherosclerotic lesion formation as compared to that of the NC group (*P *<* *.05). The HF + FA and HF + RAPA groups had decreased areas of atherosclerosis as compared to the area of atherosclerotic lesions seen in the HF group (*P *<* *.05). H&E staining of the aortic arch sections showed areas affected by atherosclerosis. As shown in Figure 2C and D, H&E staining showed that LDLR−/− mice in the HF group had a significantly increased area of atherosclerotic lesion formation compared to that of the NC group. Additionally, the HF + FA and HF + RAPA groups showed decreased areas of atherosclerotic lesions in comparison with those seen in the HF group (*P *<* *.05). These results indicate that FA attenuated the formation and progression of atherosclerotic lesions in LDLR−/− mice fed a high‐fat diet.

### 3.4 Folic acid supplementation inhibited VSMC dedifferentiation in vivo

**Figure 3:**
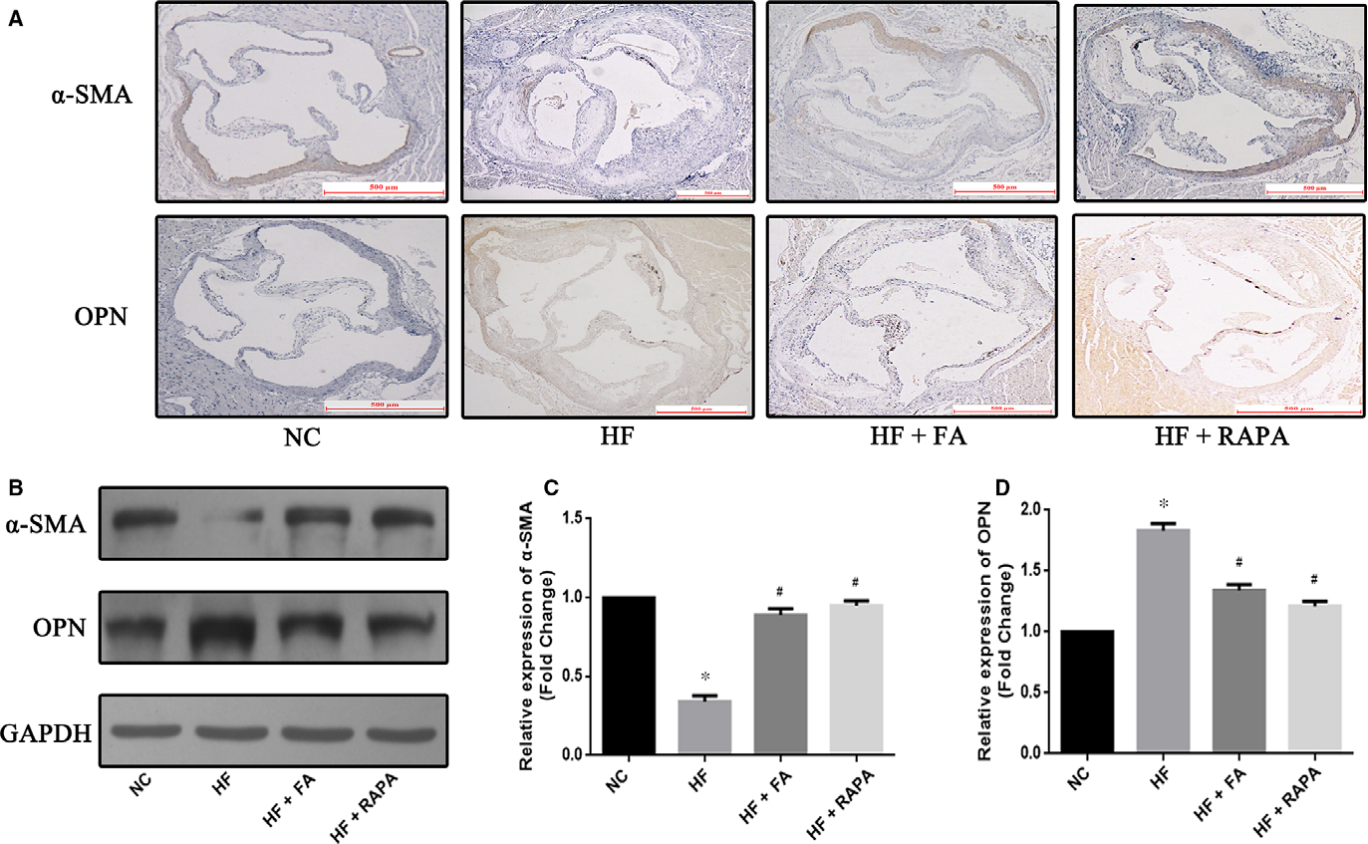
Effect of folic acid on vascular smooth muscle cell dedifferentiation. (A) Representative immunohistochemistry images showing protein expression of α‐smooth muscle actin (SMA) and osteopontin (OPN) in the thoracic aorta; (B) (C) (D) Western blot analyses show the expression of α‐SMA and OPN, and quantifiable results of Western blot analyses. Scale bar = 500 μm. The values are expressed as the mean±standard error of the mean (n = 5 for each group). *P < .05 vs the NC group; ^#^ *P* < .05 vs the HF group

To investigate the effect of FA on phenotypic switching of VSMCs, protein markers of dedifferentiation α‐SMA (a marker of contractile smooth muscle cells) and OPN (a marker of synthesis in smooth muscle cells) were detected by immunohistochemical analysis and Western blot. Immunohistochemical analysis showed that the HF group had a decreased α‐SMA expression and an increased OPN expression in aortic tissue in comparison with that seen in the NC group. However, FA supplementation increased α‐SMA expression and decreased OPN expression in the aortic tissues of LDLR−/− mice compare to that seen in the HF group (*P *<* *.05) (Figure 3A and B). Additionally, Western blot revealed up‐regulation of the expression of α‐SMA and down‐regulation of the expression of OPN in the HF + FA and HF + RAPA groups compared with that seen in the HF group (Figure 3C and D). These results suggest that FA prevents VSMCs dedifferentiation.

### 3.5 Folic acid supplementation suppressed atherosclerosis progression and VSMC dedifferentiation through the mTOR/p70S6K signalling pathway

**Figure 4:**
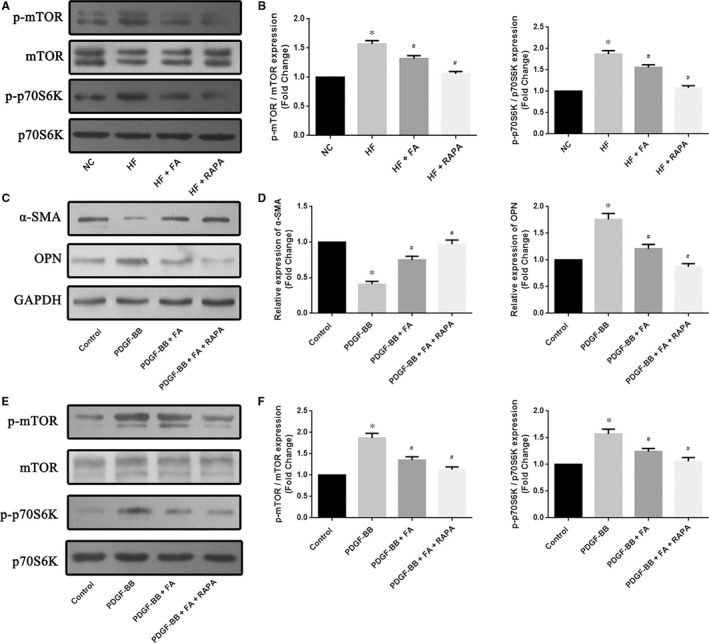
Effect of folic acid on the mTOR/p70S6K signalling pathway in vivo and in vitro. (A) (B) Western blot analyses show the expression of the phosphorylation of mammalian target of rapamycin (mTOR) and p70S6K in vivo, and quantifiable results of Western blot analyses; (C) (D) Western blot analyses show the expression of α‐SMA and osteopontin (OPN) in vascular smooth muscle cells (VSMCs), and quantifiable results of Western blot analyses; (E) (F) Western blot analyses show the expression of the phosphorylation of mammalian target of rapamycin (mTOR) and p70S6K in vitro, and quantifiable results of Western blot analyses. The values are expressed as the mean±standard error of the mean (n = 5 for each group). *P < .05 vs the NC group; ^#^ *P* < .05 vs the HF group

To elucidate whether FA regulates the phenotypic switching of VSMCs through modulation of the mTOR/p70S6K signalling pathway, we measured the phosphorylation of mTOR and p70S6K by Western blot. Compared with the NC group, the expression of p‐mTOR and p‐p70S6K was increased in the aortas of the HF group compared to that seen in the NC group (*P *<* *.05). However, both the HF + FA and HF + RAPA groups showed a decreased p‐mTOR and p‐p70S6K expression in LDLR−/− mice aortic tissue compared with that seen in the HF group (*P *<* *.05) (Figure 4A and B). In addition, FA increased α‐SMA expression and decreased OPN expression, decreased p‐mTOR and p‐p70S6K expression in VSMCs (Figure 4C, D, E and F). These results suggest that FA inhibits VSMC dedifferentiation through suppression of the mTOR/p70S6K signalling pathway.

## 4 DISCUSSION

In this study, we demonstrated that FA supplementation reduced blood lipid levels and markers of oxidative stress and inflammation in LDLR−/− mice. In addition, FA supplementation decreased the area of atherosclerotic lesions in LDLR−/− mice in comparison with LDLR−/− mice fed a high‐fat diet without FA supplementation. Additionally, we found that FA inhibited the dedifferentiation of VSMCs in vivo and in vitro. Moreover, FA may inhibit VSMC dedifferentiation through the mTOR/p70S6K signalling pathway. Overall, these findings suggest a potential mechanism for the cardioprotective effects of FA.

FA is known to have beneficial cardioprotective effects. Li et al^23^ reported that FA reduced mortality in hypertensive patients with heavy proteinuria without a history of cardiovascular disease. Guo et al^15^ found that FA therapy could significantly improve endothelial function. Most studies of the relationship between FA and atherosclerosis have focused on FA's ability to reduce serum homocysteine levels 24 or on FA's antioxidant,^7^ anti‐inflammatory^11^ and antithrombotic effects.^14^ Little is known regarding the regulatory effects of FA on VSMC dedifferentiation, which is necessary for the development and progression of atherosclerosis. Therefore, we evaluated the effects of FA on VSMC dedifferentiation and found that FA delays atherosclerosis lesion development and inhibits VSMC dedifferentiation through the mTOR/p70S6K signalling pathway in LDLR−/− mice.

High plasma concentrations of cholesterol, and in particular LDL, are one of the principal risk factors for the development of atherosclerosis.^25^ In addition, lower LDL concentrations and increased HDL levels are known to prevent the development and progression of atherosclerosis.^26^ Hyperlipidaemia causes endothelial injury and inflammation and triggers atherosclerosis.^27^ Therefore, lowering lipid levels could delay the development of atherosclerosis. In addition, prior reports have shown that FA supplementation effectively prevented atherosclerosis and reduced the concentration of serum TC.^28^ Our results are consistent with those of previous studies that showed that FA supplementation significantly reduced serum levels of TC, TG, LDL‐C and VLDL‐C and increased HDL‐C. Similar results have been seen with the administration of rapamycin. Rapamycin, a known mTOR inhibitor, increased serum concentrations of HDL‐C and decreased concentrations of TG, TC and LDL‐C and reduced lipid deposition in atherosclerotic mice fed a high‐fat diet.^29^, ^30^ The present study compared the results of rapamycin administration to those of FA supplementation in LDLR−/− mice fed a high‐fat diet. Furthermore, FA treatment substantially decreased lipid deposition and effectively decreased the atherosclerotic lesions in LDLR−/− mice.

Previous studies have shown that oxidative stress and inflammation play a critical role in the development of atherosclerosis.^31^, ^32^ A high‐fat diet has been shown to increase oxidative stress and arterial inflammation.^33^ Other studies have shown that FA has antioxidant^7^ and anti‐inflammatory properties.^11^ Carnicer et al demonstrated that FA supplementation can decrease atherosclerotic lesions in apoprotein (Apo)E‐deficient mice independent of plasma homocysteine and cholesterol levels. They also noted increased apolipoprotein AI, AIV and B levels and decreased oxidative stress.^31^ Also, Tousoulis et al^20^ demonstrated that FA's anti‐inflammatory effect was associated with a parallel improvement in lipid levels and an attenuation of atherosclerotic lesion progression. Moreover, it is well known that the use of the mTOR inhibitor, rapamycin, also reduces oxidative stress, modulates the inflammatory response and attenuates atherosclerotic lesion progression in ApoE−/− mice.^34^ Our findings confirm those of these previous studies and again demonstrate that FA prevents oxidative stress, decreases the levels of inflammatory cytokines and ameliorates the progression of atherosclerosis in high‐fat‐fed LDLR−/− mice.

Mature VSMCs retain phenotypic plasticity, and VSMCs constitute the main component of atherosclerotic plaques.^35^ Previous studies have demonstrated that VSMC dedifferentiation is a pathophysiological process fundamental for the progression of various arterial diseases.^36^ The prevention of phenotypic switching of VSMCs has been shown to delay the progression of atherosclerosis.^2^, ^36^ Studies by several investigators, including the present study, have shown that a high‐fat diet can induce VSMC dedifferentiation.^20^, ^31^ Recently, Hasanov et al^37^ observed that endosialin may be a relevant regulator of phenotypic VSMC remodelling and reduced atherosclerosis in vivo. Ding et al^38^ confirmed that 5′‐AMP‐activated protein kinase catalytic subunit alpha‐2 plays a protective role in suppressing VSMC dedifferentiation and in enhancing plaque stability in advanced atherosclerosis. These results indicate that inhibition of VSMC phenotypic switching can retard atherosclerotic plaque progression. In agreement with these previous studies, the present study shows that FA inhibits VSMC dedifferentiation in vivo and in vitro. Our immunohistochemical results show that FA supplementation increased α‐SMA expression and decreased OPN expression in LDLR−/− mouse aortic tissue. Consistent with these immunohistochemical results, Western blotting showed that FA ameliorated phenotypic switching in VSMCs from atherosclerotic plaques in LDLR−/− mice. Additionally, our previous studies have found that FA can decrease lipid deposition, oxidative stress and inflammation. Therefore, we speculate that FA, through decreases in lipid levels, oxidative stress and inflammation, can inhibit phenotype switching of contractile‐type VSMCs to synthesis‐type VSMCs.

mTOR/p70S6K signalling has been reported to function as a critical regulator in the development of atherosclerosis, and the inhibition of mTOR has reportedly suppressed the development of atherosclerosis. Therefore, the mTOR signalling pathway has been identified as a therapeutic target.^39^, ^40^ Rapamycin and its analogues are widely used in preventing restenosis and suppressing atherosclerosis.^41^ Jahrling et al^21^ showed that rapamycin‐mediated mTOR attenuation decreased the atherosclerosis lesion area in high‐fat‐fed LDLR−/− mice. In the present study, we demonstrated that FA ameliorated atherosclerosis progression via suppression of the mTOR/p70S6K signalling pathway. Furthermore, studies have shown that the mTOR/p70S6K signalling pathway is involved in VSMC dedifferentiation.^42^ Ding et al^43^ reported that adiponectin suppressed VSMC dedifferentiation via inhibition of mTOR C1 and its effector S6 kinase 1 (S6K1). Additionally, Zhang et al^44^ demonstrated that miR‐99a inhibited insulin‐induced VSMC dedifferentiation via the inhibition of mTOR signalling. In the present study, we demonstrated that FA could suppress the mTOR/p70S6K signal pathway and inhibit VSMC dedifferentiation in vivo, and we found that FA through inhibiting the mTOR pathway inhibits VSMC dedifferentiation, results consistent with our previous in vitro studies.^45^ However, the regulation of the mTOR/p70S6K signalling pathway and its effects on the phenotypic switching of VSMCs in atherosclerosis is still not fully understood. Recent studies have demonstrated that mTOR signalling regulates many metabolic and physiological processes, including lipid metabolism,^46^ oxidative stress^40^ and inflammation.^30^ Therefore, we suggest that the possible effects by which FA reduces VSMC dedifferentiation are related to the modulating mTOR/p70S6K signalling pathway effects of FA, including a reduction in lipid levels and inhibition of oxidative stress and inflammation. These mechanisms may be involved in the beneficial metabolic and cardiovascular effects of FA supplementation.

In conclusion, FA showed important beneficial cardioprotective effects in high‐fat‐fed LDLR−/− mice. This study provides evidence for the role of FA in regulating VSMC phenotypic switching, decreasing blood lipids, preventing oxidative stress and decreasing the levels of inflammatory cytokines in atherosclerotic lesions. All of these effects of FA may represent mechanisms underlying the anti‐atherosclerotic actions of FA observed in vivo. Our research may enhance the understanding of VSMC phenotypic switching and suggest potential therapeutic or preventive targets for the treatment of patients with cardiovascular diseases.

## CONFLICT OF INTEREST

All of the authors declare that they have no conflict of interests regarding the contents of this article.
